# Serum folate levels in bipolar disorder: a systematic review and meta-analysis

**DOI:** 10.1186/s12888-019-2269-2

**Published:** 2019-10-22

**Authors:** Yung-Chi Hsieh, Li-Shiu Chou, Ching-Hua Lin, Hung-Chi Wu, Dian-Jeng Li, Ping-Tao Tseng

**Affiliations:** 1 Kaohsiung Municipal Kai-Syuan Psychiatric Hospital, No.130, Kaisyuan 2nd Rd., Lingya Dist, Kaohsiung City, 802 Taiwan; 2 WinShine Clinics in Specialty of Psychiatry, Kaohsiung City, Taiwan; 3 Prospect Clinic for Otorhinolaryngology & Neurology, Kaohsiung City, Taiwan

**Keywords:** Folate, Folic acid, Biomarker, Meta-analysis, Bipolar disorder

## Abstract

**Background:**

Bipolar disorder (BD) is a major psychiatric illness, however its physiopathology is unclear. The role of folate in the physiopathology of BD is controversial. We conducted this systematic review and meta-analysis to investigate the effect of folate in BD patients.

**Methods:**

We performed a thorough literature study of the PubMed, Embase, ScienceDirect, ClinicalKey, Cochrane Library, ProQuest, Web of Science, and ClinicalTrials.gov databases until December 21st, 2018. Random effects meta-analysis was conducted.

**Results:**

Six articles involving 481 patients with BD and 760 controls were included. The meta-analysis results suggested that serum folate levels in the patients with BD were significantly lower than those in the controls (Hedges’ g = − 0.211, 95% confidence interval = − 0.391 to − 0.031, *p* = 0.021).

**Conclusion:**

The current meta-analysis show it might be association between lower serum folate levels and patient with BD. However, we could not distinguish the potentially confounding effects of mood states on the folate levels. Further prospective studies including subjects with different mood states and possible physiopathology are warranted to investigate the association between folate deficiency and the etiology of BD.

## Highlight

1. Folate is a key element in neurotransmitter synthesis and had been found to be abnormally synthesized in numerous psychiatric diseases.

2. The current meta-analysis showed that serum folate levels in individuals with bipolar disorder (BD) were lower than in healthy controls.

3. To provide clearer information about the role of folate in physiopathology of BD, future investigations should be warranted.

## Background

Bipolar disorder (BD) is a major health concern due to the difficulty in achieving complete remission [1, 2]. It has been reported that up to two-thirds of patients diagnosed with BD have moderate to severe disease, [3] and that more than half of patients experience recurrence [4, 5] or subthreshold episodes [6] despite regular treatment. Furthermore, the symptoms of BD may negatively affect social-economic performance and interpersonal relationships among BD patients, [7, 8] and it is associated with a predominantly functional impairment, lower quality of life, and death due to suicide or comorbid physical diseases [9, 10]. Accordingly, BD is the sixth leading cause of disability globally [11]. Studies focusing on the physiology of BD may help to elucidate its etiology and lead to new treatment strategies.

In an effort to understand the physiopathology underlying BD, researchers have attempted to identify potential biomarkers for patients with BD and apply the new field of “precision psychiatry.” [12–14] Neurotrophic factors such as brain-derived neurotrophic factor (BDNF) [15, 16] and transforming growth factor beta 1 (TGF-β1) [17] have been proposed as potential peripheral biomarkers in BD. In addition, purinergic abnormalities leading to uric acid levels variations have been recently reported in BD [18, 19]. Furthermore, BD has been associated with a poor nutritional status, [20, 21] as patients with BD have been reported to be more likely to consume unhealthy prepared food without essential vitamins, minerals and n–3 fatty acids [22].

Folate is a water-soluble vitamin (Vit. B9), which is vital for cellular activity and immune and nervous system functions. Folate can reduce the level of homocysteine and act as a precursor of methionine and S-adenosyl methionine, which are major sources of methyl groups in the brain [23]. Folate has also been reported to be involved in the synthesis of neurotransmitters and many other metabolic pathways, which may be associated with exacerbations of psychiatric disorder [24, 25]. Folate is also essential for deoxyribonucleic acid (DNA) repair, and it has been intimately associated with methylation and the formation of neurotransmitters in the central nervous system, such as serotonin [26–28]. Previous studies have investigated the association between levels of folate and BD. A cross-sectional study indicated that the mean folate level in patients was significantly lower than in controls, and a positive correlation was found between depression and low folate levels [29]. However, another study including patients with manic episodes demonstrated no significant differences in folate level between the patients and healthy control [30]. In addition, a clinical trial on biomedical predictors of treatment response in patients with bipolar depression also found no significant difference in folate level between patients who received lamotrigine and controls [31].

The inconsistencies in the results of differences in folate levels between patients with BD and healthy controls raise a crucial question about which level of folate may exert an effect on exacerbations of BD. A previous systematic review and meta-analysis reported that low folate status was associated with depression [32]. Decreased folate levels have been also proposed as possible biomarker of schizophrenia, according to a recent review [33]. However, there is still a need for systematic reviews or meta-analyses summarizing previous literature with regards to BD. Therefore, we conducted a systematic review and meta-analysis to investigate the effect of folate on patients with BD, and to explore differences in folate levels between patients with BD and healthy subjects. We hypothesized that the level of folate among patients with BD may be lower than that among healthy individuals, which might reflect the potential physiopathology of BD.

## Methods and materials

We complied with the Preferred Reporting Items for Systematic Reviews and Meta-Analyses (PRISMA) guidelines [34] (see Additional file 1 and Additional file 2). The current meta-analysis followed the protocol which was not published a priori.

### Eligibility criteria

The inclusion criteria were: (a) studies in humans; (b) observational studies, including a cross-sectional or cohort study design; (c) investigating the peripheral levels of folate between patients diagnosed with BD and those of the healthy controls; (d) the mood status investigated could be manic episode, depressive episode, euthymic episode, or mixed episode; (e) BD patients with/without concomitant medication; (f) the peripheral folate could be that of serum, plasma, or whole blood; and (g) formal published studies in journal articles.

The exclusion criteria consisted of (a) non-human studies; (b) non-clinical studies, such as review articles or case reports; (c) not formal published studies in journal articles, such as meeting abstracts or posters; and (d) not investigating peripheral folate levels in BD patients and controls.

### Search strategy and study selection

Two well-trained authors (DJ Li and YC Hsieh) conducted a systematic literature search from inception until December 21st, 2018 with the following keywords: “(vitamin B12 OR cobalamin OR folate OR folic acid OR folacin OR vitamin B9 OR homocysteine OR methionine) AND ((bipolar depression) OR (bipolar mania) OR (mania) OR (bipolar disorder))” on PubMed, Embase, ScienceDirect, ClinicalKey, Cochrane Library, ProQuest, Web of Science, and ClinicalTrials.gov platforms for all eligible studies. To include as many studies as possible, we did not set any limitation in language (Additional file 1: Table S3). We were also hand-searched the reference lists of the included articles and recent reviews to identify more articles [35–39].

The titles and abstracts of all results for potential eligibility screened by the two authors (DJ Li and YC Hsieh) independently. A final list of included studies was achieved after both authors reviewed the full-text articles of potentially eligible papers. A third reviewer (PT Tseng) helped to resolve inconsistencies through discussion.

### Outcome settings

The outcome was the difference in peripheral folate levels between patients with BD and control groups (calculated as Hedges’ *g* statistic and corresponding 95% confidence intervals (CIs) and *p* values). Because of presumed differences in the tools used to detect folate among the studies, we did not choose differences in means as the effect sizes (ESs) of our primary outcome. The control groups were defined as those without BD or any other psychiatric illness. We preferred to choose control groups consisting of healthy (asymptomatic) subjects, if data were available. We contacted the primary authors to request the original data, when data were not available from the included studies. We contacted the authors via email on two conditions if necessary (a second email was sent a week later if no response was received following the initial email). Of all the papers we found, if there was no relevant data concerning folate levels, we attempted to use another compatible statistical parameter (e.g. *p* value and sample size) to estimate the ESs according to the protocol in the Comprehensive Meta-Analysis manuals and guidance on the Comprehensive Meta-Analysis website [40] to convert and pool the ESs into Hedges’ *g*.

### Methodological quality appraisal

The Newcastle-Ottawa scale (NOS) was used to assess the quality of the included studies. In short, the NOS was based on a version previously used in a meta-analysis study published in the British Journal of Psychiatry in 2013, of which scores ranging from zero to sixteen (Additional file 1: Table S1) [41]. The NOS were accessed by the two authors (DJ Li and YC Hsieh) independently. A third reviewer (PT Tseng) helped to resolve inconsistencies through discussion.

### Meta-analysis procedure

With regard to the anticipated heterogeneity in the basic study population, we conducted the meta-analyses with a random effects model. As mentioned above, the primary ES was estimated as Hedges’ *g* with 95% CIs to compare folate levels in patients with BD versus controls.

### Sensitivity test, heterogeneity, publication bias, and meta-regression

Sensitivity testing with the “leave-one-out sensitivity analysis” test was used to evaluate whether or not the results of the meta-analysis was caused by any outliers within the recruited studies [42]. Heterogeneity was assessed using the Cochrane Q test and its corresponding *p* value [43]. We conducted meta-regression analyses with unrestricted maximum likelihood random-effects when data on each potential moderator was provided by at least ten different studies. We assessed publication bias through the inspection of funnel plots [44] and with the Egger’s regression test [45]. We used the Duval and Tweedie’s trim-and-fill procedure to adjust the potential publication bias, when evidence of publication bias was found [46]. The current meta-analyses were performed using the Comprehensive Meta-Analysis software, version 3 (Biostat, Englewood, NJ). The threshold for statistical significance was set at a two-tailed *p*-value < 0.05.

## Results

### Study selection

**Fig. 1:**
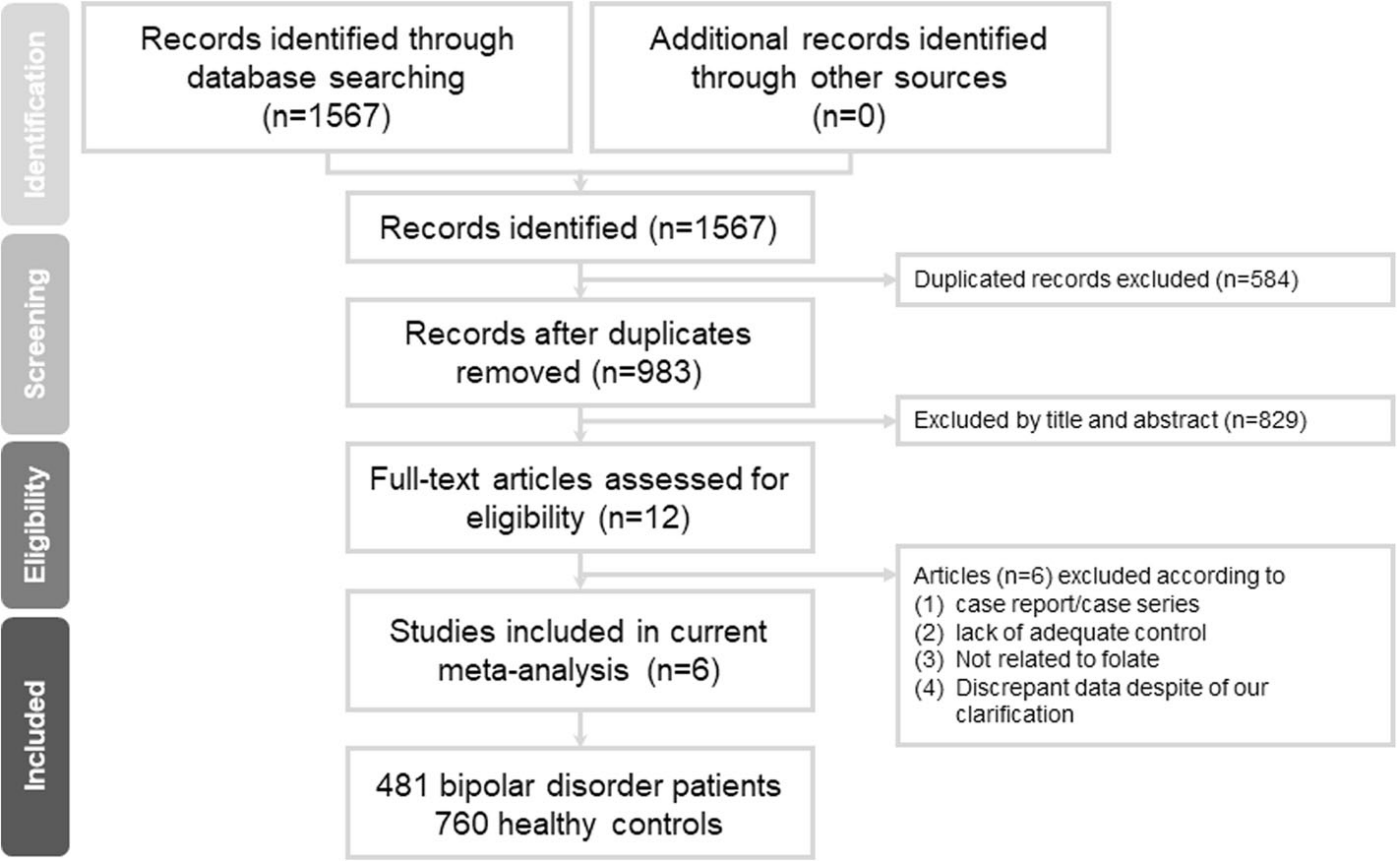
the flowchart of the current meta-analysis

Figure 1 is the summary of details of the search results. In brief, a total of 12 studies were selected at the full-text review stage, of which 6 were excluded for a variety of reasons (see Fig. 1). Additional file 1: Table S2 presented a list of the excluded articles. In total, six articles met the inclusion criteria, and their details are summarized in Tables 1 and 2 [29, 47–51].

**Table 1:** Characteristics of the included studies

Author (year)	Study design	Criteria	bipolar subtype	symptom severity	Mood state and subjects’ characteristics	n (male/female)	Mean age	Source and assay	Comorbidities and exclusion criteria
Doganavsargil Baysal, G.O. (2013)	case-control study	DSM-IV-TR	bipolar I disorder	maintain	Euthymic patient Healthy control	60 (n/a) 20 (n/a)	n/a	Serum, n/a	Patients who had neurologic diseases, cardiovascular diseases, renal diseases, diabetes mellitus, hypo/hyperthyroid- ism, non-psychotropic drug use that could affect HCY levels, substance abuse, an IQ score below 80 based on the WAIS-R test (Epir and Iskit, 1972), a history of electroconvulsive therapy during the previous 6 months, or patients who tested positive for biochemical markers suggestive of abnormal thyroid, liver or renal functions were not included in the study.
Ezzaher, A. (2011)	case-control study	DSM-IV	bipolar I disorder	n/a	Depressive, manic, euthymic patient Healthy control	92 (62/30) 170 (79/91)	36.0 ± 11.1 43.7 ± 14.2	Serum, microparticle enzyme immunoassay on Elecsys 2010	The exclusion criteria were age < 18 years, other psychiatric illnesses, epilepsy, mental retardation, hormone therapy, history of vitamin use, diabetes, cardiovascular disease, thyroid dysfunction, liver disease, or renal dysfunction.
Diass, V.V. (2009)	case-control study	DSM-IV	bipolar I disorder	maintain	Euthymic patient Healthy control	65 (24/41) 49 (14/35)	37.8 ± 10.5 33.6 ± 9.8	Serum, electrochemiluminescence immunoassay	Patients with neurological disorders, previous head trauma, physical illnesses requiring medical intervention, substance abuse, or an ECT course in the preceding 6 months were excluded.
Ozbek, Z. (2008)	case-control study	DSM-IV	Bipolar disorder	n/a	Unspecified mood patient Healthy control	197 (74/123) 238 (67/171)	40.6 ± 12.2 41.3 ± 12.7	Serum, electrochemiluminescence immunoassay	None of the subjects had significant neurological comorbidity, epilepsy, mental retardation or a history of substance abuse. None of the subjects presented with current or past history of cardiovascular disease, endocrinological and metabolic disease, or a family history of coronary heart disease.
Lerner, V. (2006)	case-control study	DSM-IV	bipolar I disorder	acute	Mania, inpatient Healthy control	22 (n/a) 250 (156/94)	n/a 42.17 ± 14.2	Serum, Electrochemiluminescence	n/a
Hasanah, C.I. (1997)	case-control study	DSM-III-R	bipolar I disorder	acute	Mania, inpatient Healthy control	45 (24/21) 33 (13/20)	30.0 29.0	serum, the IMx system	The exclusion criteria were, mood incongruent psychotic features, concomitant medical illness, substance misuse, pregnancy, puerperium and those on drug treatment. Those with noticeable weight loss by close relatives were also excluded.

Abbreviation: *DSM-IV-TR* Diagnostic and Statistical Manual of Mental Disorders, 4th edition, Text-Revision; *DSM-IV* Diagnostic and Statistical Manual of Mental Disorders, 4th edition; *DSM-III-R* Diagnostic and Statistical Manual of Mental Disorders, 3rd edition, Revision; *n/a* not available

**Table 2:** Baseline characteristics of the recruited subjects in the individual studies

Author (year)	psychotropic medication use	proportion of specific medication	Healthy Control group criteria	dietary habit	nutritional status	genetic characteristics	Country
Doganavsargil Baysal, G.O. (2013)	yes	lithium: 11 patients (18.33%) valproic acid:6 patients (10%) mood stabilizing drugs plus antipsychotic drugs:30 patients (50%) two mood stabilizing drugs: 12 patients (20%)	Subjects who had not been diagnosed with a psychiatric disease and did not have a known significant medical condition or mental development disorder	n/a	n/a	n/a	Turkey
Ezzaher, A. (2011)	yes	Valproic acid: 46 patients (50%) Lithium: 10 patients (10.9%) Carbamazepine: 9 patients (9.8%) Valproic acid and lithium: 3 patients (3.2%) Antipsychotics: 24 patients (26.1%)	None of the controls had psychiatric illnesses, epilepsy, mental retardation, hormone therapy, history of vitamin use, diabetes, cardiovascular disease, thyroid dysfunction, liver disease or renal dysfunction.	n/a	n/a	C677T MTHFR polymorphism was studied but no significant finding	Tunisia
Diass, V.V. (2009)	yes	n/a	Healthy individuals were screened for personal and first-degree family axis I psychiatric disorders with the MINI.	n/a	n/a	n/a	Spain
Ozbek, Z. (2008)	yes	Atypical antipsychotic: 17% typical antipsychotic: 65% atypical antipsychotic in combination with typical antipsychotic: 18%	Healthy and unrelated volunteers without psychiatric disorders were selected as a control group.	n/a	n/a	Patients carrying TT and/or AA and AC genotypes in methylenetetrahydrofolate (MTHFR) gene variants of c.1298A > C (Glu429Ala) and c.677C > T (Ala222Val) reduced folate levels	Turkey
Lerner, V. (2006)	yes	n/a	Subjects from community clinics with no record of psychiatric illness	n/a	n/a	n/a	Israel
Hasanah, C.I. (1997)	inpatient, n/a	n/a	The control group was taken from the out-patient clinic. Only patients with minor ailments like headaches, cough and cold were taken. Exclusion criteria for the control group were, family history and past history of psychiatric illness, substance misuse, recent weight loss, sleep disturbance, pregnancy and puerperium.	n/a	n/a	n/a	Malaysia

Abbreviation: *n/a* not available

### Characteristics and methodological quality of the included studies

All of the included studies provided comparisons of differences in serum folate levels between patients with BD and controls (BD group: *n* = 481, mean age = 37.9 years, mean female proportion = 56.1%; controls without BD: *n* = 760, mean age = 41.1 years, mean female proportion = 55.5%). None of the included studies compared peripheral folate levels in plasma or whole blood. Of these six studies, two [29, 50] measured folate levels in patients with mania, two [49, 51] in patients with euthymia, and two [47, 48] did not specify the mood state. Of these six studies, two [47, 48] consisted of patients on medication, and the other four studies did not mention information about medication.

In all of the recruited studies, the diagnosis of BD was made according to the Diagnostic and Statistical Manual of Mental Disorders (DSM) [52]. The assays used to detect folate levels included chemiluminescent immunoassay, microparticle enzyme immunoassay, electrochemiluminescence immunoassay, solid phase radioassay, and the IMx system (Table 1). Concerning the methodological quality of the included studies, the median NOS score was 8 with 25–75% quantile 7.25–8 (Additional file 1: Table S1).

### Main results of the current meta-analysis

**Fig. 2:**
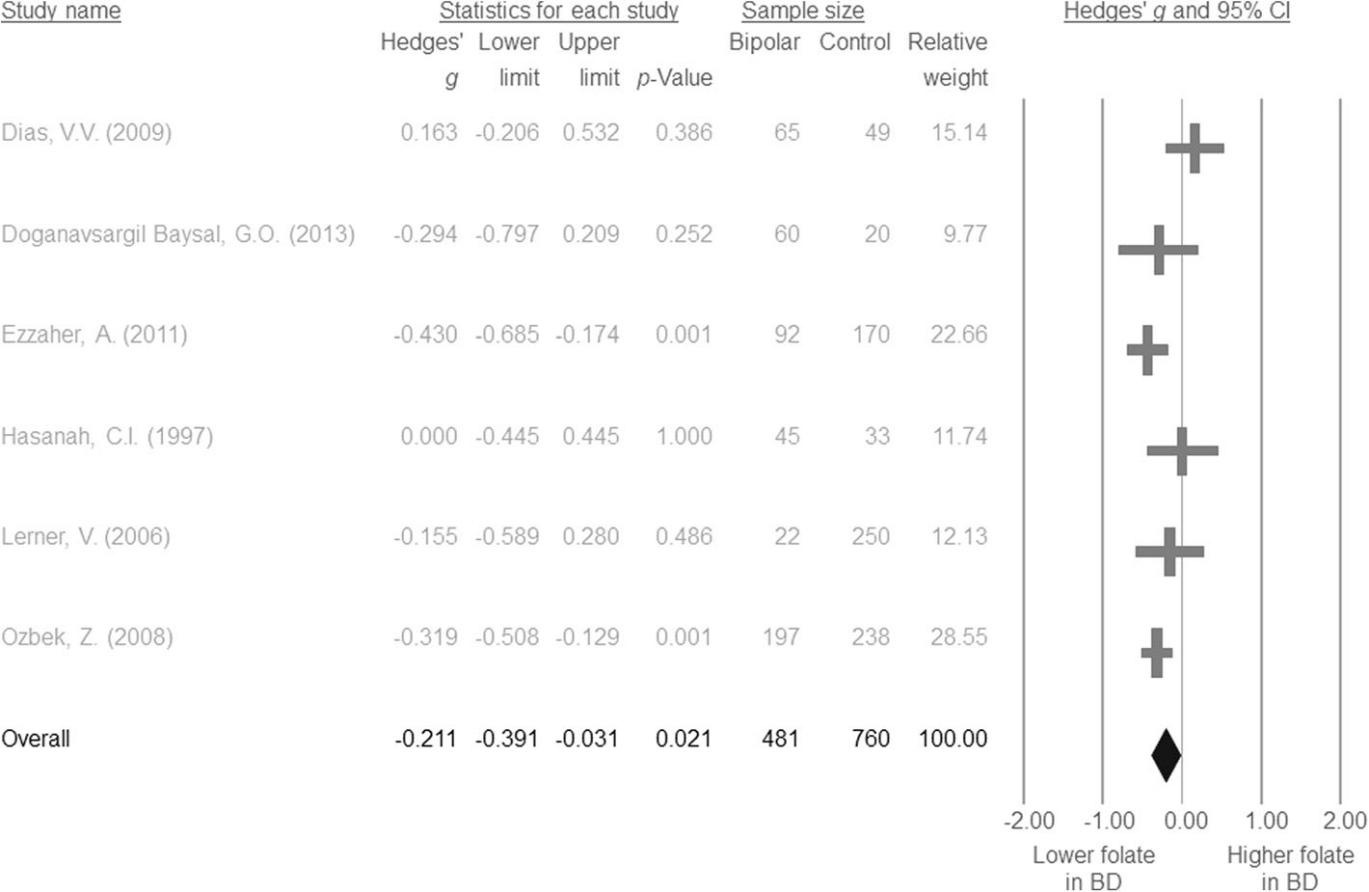
Forest plot of the current meta-analysis of serum folate levels in patients with bipolar disorder and controls. Figure 2 indicated the significantly lower serum folate levels in the BD patients than that of controls (Hedges’ *g* = − 0.211, 95% CI = -0.391 to − 0.031, *p* = 0.021). Abbreviation: BD: bipolar disorder; CI: confidence interval

The results of the meta-analysis indicated that serum folate levels in the patients with BD were significantly lower than those in the controls (*k* = 6, Hedges’ *g* = − 0.211, 95% CI = -0.391 to − 0.031, *p* = 0.021) (Fig. 2) without significant evidence of heterogeneity (Q value = 8.647, df = 5, *I*^*2*^ = 42.177%, *p* = 0.124) or publication bias via inspection of the funnel plot (Additional file 2: Figure S1) and Egger’s regression test (t = 1.428, df = 4, *p* = 0.226).

### Sensitivity test

The significant results of the meta-analysis did not become insignificance after removing any one of the included studies, except for the study by Ezzaher (2011, 47] (after removal of that study, Hedges’ *g* = − 0.149, 95% CI = -0.349 to 0.051, *p* = 0.143) or that by Ozbek (2008) (after removal of that study, Hedges’ *g* = − 0.160, 95% CI = -0.401 to 0.082, *p* = 0.195) [48].

### Meta-regression

The meta-regression could not be performed because of lack of sufficient datasets.

## Discussions

To our knowledge, the current meta-analysis is the first study specifically focusing on the differences in folate levels between patients with BD and healthy controls. The current meta-analysis indicated that the patients with BD had significantly lower serum folate levels than the controls.

Our results showed significantly lower folate levels in the patients with BD. There are two potential explanations for this finding. First, due to the biological role of folate, the biologically active form of folate, L-methylfolate, may act as a trimonoamine modulator and enhance the synthesis of three monoamines including dopamine, norepinephrine, and serotonin, and this has been reported to be involved in the efficacy of antidepressants [53]. The significantly lower folate level in the patients with BD could have been the result of dysregulation of methylenetetrahydrofolate reductase (MTHFR) enzyme, which is a product of the MTHFR gene [54]. A previous study also reported the role of MTHFR gene polymorphisms in the association between a low folate level and BD, [55] and another study reported that this was a potential risk factor for the development of BD [48]. Furthermore, a meta-analysis of MTHFR gene variants also provided evidence of epigenetic involvement in the pathophysiology of BD [56].

The other potential explanation may be due to malnutrition, as patients with BD have been reported to have deficient folate intake [57]. In an epidemiological and cross-sectional survey, patients with BD had significantly poor dietary habits and malnutrition status [58]. Furthermore, patients with BD tend to consume unhealthy prepackaged or prepared food, which lacks sufficient levels of essential vitamins [22]. Another cross-sectional study also indicated a lower folate intake than the recommended daily allowance among patients with BD and major depressive disorder [57]. Following the rationale above, previous review based on randomized controlled studies provided the evidences about the benefit of folate supplement in manic patients [59]. Therefore, we suggest that clinicians should pay special attention to the nutritional status, including folate intake, of patients with BD which would be of clinical importance.

The relationship between folate levels and mood status had been widely explored, especially in patients with depressive mood [32]. The previous study of this relationship might be one possible explanation of our findings of lower folate levels in the patients with BD, which consisted of depressive, manic, and mixed mood. However, due to few studies included, we could not perform further analysis to distinguish the relationship with folic acid and specific mood status.

### Limitations

Several limitations should be addressed for the current meta-analysis. First, the overall meta-analytic results became insignificant during sensitivity test, which was not only due to the small sample sizes but also the small effect sizes. The possibility of both type I and type II errors cannot not be excluded. Also, an effect size around 0.2 was small, [60] which might not be relevant to clinically significant. Second, we could not perform sub-group analysis due to the limited number of comparison arms, such as different mood states of BD. Therefore, at current stage, we could not distinguish the potential effect of mood states on the folate levels. Third, because the association between BD and folate levels had not been well-established, there were only few clinical studies addressing this issue. Therefore, we could not perform further investigations into the dietary habit, nutritional status, prescription of psychotropic agents, and genetic characteristics because of too few studies to make subgroup analysis of the included BD patients because of a lack of data. Fourth, most of the included studies were cross-sectional studies, and progressive changes in folate levels could not be estimated. Fifth, the prescribed mood stabilizers and antipsychotics may have had a cofounding effect on the level of folate in the patients with BD [61], and this effect was not controlled in the included studies. Sixth, the power of the Egger’s test with substantially fewer than 10 studies is not recommended [62]. Finally, as the natural limitation of meta-analysis, we could only observe a phenomenon but not explore the pathophysiology behind it.

## Conclusion

The current meta-analysis show that there might be association between lower serum folate levels and patient with BD. However, we could not distinguish the potentially confounding effects of mood states on the folate levels. Future investigations on potential differences in folate levels in BD patients with different mood states, and the potential physiopathology behind the folate deficiency and the etiology of BD should be warranted.

## Supplementary information

**Additional file 1: Table S1.** Newcastle-Ottawa Scale (NOS) of recruited studies, **Table S2.** Excluded studies and reasons and **Table S3.** Database and keyword search strategy.

**Additional file 2: Figure S1.** Funnel plot of current meta-analysis.

## Data Availability

Specific data sets used and/or analyzed during the current study are available from the corresponding author on reasonable request.

## Abbreviations

BD: Bipolar disorder
BDNF: Brain-derived neurotrophic factor
BMI: Body mass index
CI: Confidence interval
DNA: Deoxyribonucleic acid
DSM: Diagnostic and Statistical Manual of Mental Disorders
ES: Effect size
MTHFR: Methylenetetrahydrofolate reductase
NOS: Newcastle-Ottawa scale
PRISMA: Preferred Reporting Items for Systematic Reviews and Meta-Analyses
SD: Standard deviation
