# Supplementary material for: Serum folate levels in bipolar disorder: a systematic review and meta-analysis
Source: BMC Psychiatry. 2019 Oct 22;19:305. doi: 10.1186/s12888-019-2269-2 (PMC6805488; doi:10.1186/s12888-019-2269-2)
Supplement: Supplementary file 1 — Additional file 1: Table S1. Newcastle-Ottawa Scale (NOS) of recruited studies, Table S2. Excluded studies and reasons and Table S3. Database and keyword search strategy. [file 12888_2019_2269_MOESM1_ESM.docx]

**Supplement table 1**: Newcastle-Ottawa Scale (NOS) of recruited studies

| Author (year) | Selection | Comparability | Exposure | Total |
| --- | --- | --- | --- | --- |
| Doganavşargil Baysal, G.O. (2013) | 4 | 2 | 3 | 9 |
| Ezzaher, A. (2011) | 4 | 1 | 3 | 8 |
| Diass, V.V. (2009) | 4 | 2 | 2 | 8 |
| Ozbek, Z. (2008) | 4 | 1 | 2 | 7 |
| Lerner, V. (2006) | 4 | 2 | 2 | 8 |
| Hasanah, C.I. (1997) | 2 | 1 | 2 | 5 |

There are 3 domains and 8 questions. In each question, the scale is from 0-2, 0 is high risk of bias and 2 is low risk of bias

**Selection:**

1. Is the case definition adequate?

a) yes, with independent validation

b) yes, e.g. record linkage or based on self reports

c) no description

2.Representativeness of the cases

a) consecutive or obviously representative series of cases

b) potential for selection biases or not stated

3. Selection of Controls

a) community controls

b) hospital controls

c) no description

4. Definition of Controls

a) no history of disease (endpoint)

b) no description of source

**Comparability**

1. Comparability of cases and controls on the basis of the design or analysis

a) study controls for _______________ (Select the most important factor.)

b) study controls for any additional factor (This criterion could be modified to indicate specific

control for a second important factor.)

**Exposure**

1. Ascertainment of exposure

a) secure record (e.g. surgical records)

b) structured interview where blind to case/control status

c) interview not blinded to case/control status

d) written self report or medical record only

e) no description

2. Same method of ascertainment for cases and controls

a) yes

b) no

3.Non-Response rate

a) same rate for both groups

b) non responder described

c) rate different and no designation

**Supplement table 2:** Excluded studies and reasons

| Excluded reason | Study |
| --- | --- |
| No control subject | EM Tunbridge (2017): Biochemical and genetic predictors and correlates of response to lamotrigine and folic acid in bipolar depression: Analysis of the CEQUEL clinical trial |
|  | Andrew A. Nierenberg (2016): L-Methylfolate for Bipolar I Depressive Episodes: An Open Trial Proof-of-Concept Registry |
|  | Agnieszka Permoda-Osip (2013): Hyperhomocysteinemia in Bipolar Depression: Clinical and Biochemical Correlates |
|  | A. Coppen (1986): Folic Acid Enhances Lithium Prophylaxis |
| Discrepant statement in the manuscript despite of our contact to authors and clarification* | F Chiarani (2013): Homocysteine and other markers of cardiovascular risk during a manic episode in patients with bipolar disorder. |
| No folate data | M.W.P. Camey (1990): Red cell folate concentrations in psychiatric patients. |

*: mentioning “no significant difference between the three groups” in the texture but significantly lower folate in euthymic BD patients than in controls according to the mean/SD in table

**Supplement table 3**: Database and keyword search strategy

| Database | Keyword | Limitation | Date | Result |
| --- | --- | --- | --- | --- |
| PubMed | (folate OR folic acid OR folacin OR vitamin B9) AND ((bipolar depression) OR (bipolar mania) OR (mania) OR (bipolar disorder)) | not use | 2018/12/21 | 103 |
| Embase | (folate OR folic acid OR folacin OR vitamin B9) AND ((bipolar depression) OR (bipolar mania) OR (mania) OR (bipolar disorder)) | not use | 2018/12/21 | 420 |
| ScienceDirect | (folate OR folic acid OR folacin OR vitamin B9) AND ((bipolar depression) OR (bipolar mania) OR (mania) OR (bipolar disorder)) | research article | 2018/12/21 | 843 |
| ClinicalKey | (folate OR folic acid) AND (bipolar disorder) | not use | 2018/12/21 | 102 |
| Cochrane Library | (folate OR folic acid OR folacin OR vitamin B9) AND ((bipolar depression) OR (bipolar mania) OR (mania) OR (bipolar disorder)) | not use | 2018/12/21 | 33 |
| ProQuest | (folate OR folic acid OR folacin OR vitamin B9) AND ((bipolar depression) OR (bipolar mania) OR (mania) OR (bipolar disorder)) | not use | 2018/12/21 | 33 |
| Web of Science | (folate OR folic acid OR folacin OR vitamin B9) AND ((bipolar depression) OR (bipolar mania) OR (mania) OR (bipolar disorder)) | not use | 2018/12/21 | 33 |
