# Supplementary figures and images for: Serum folate levels in bipolar disorder: a systematic review and meta-analysis
Source: BMC Psychiatry. 2019 Oct 22;19:305. doi: 10.1186/s12888-019-2269-2 (PMC6805488; doi:10.1186/s12888-019-2269-2)

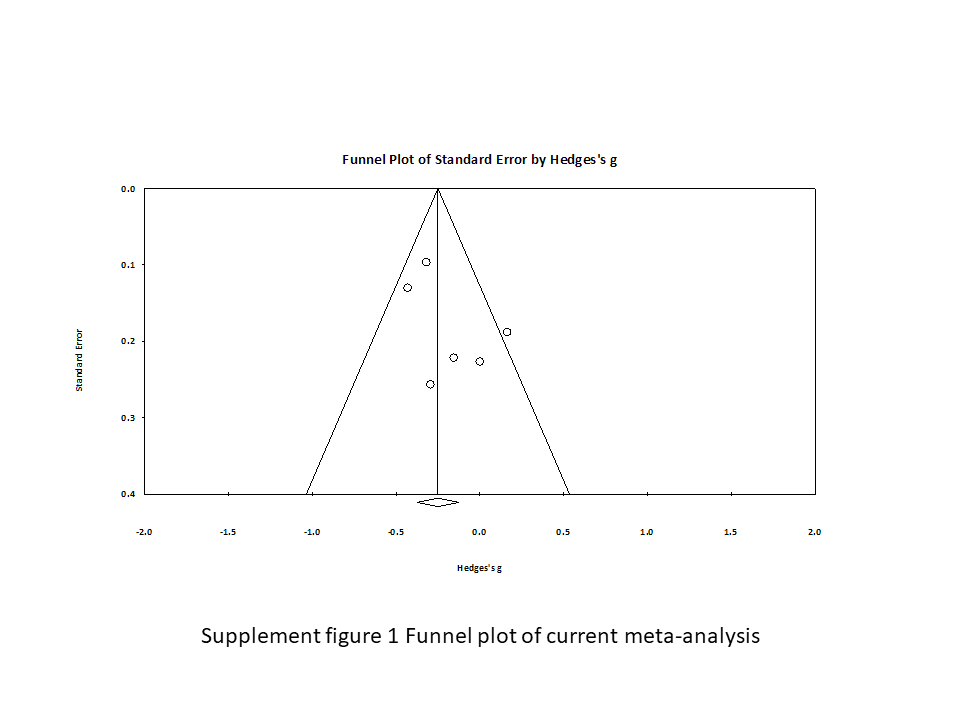

Supplement: Supplementary file 2 — Additional file 2: Figure S1. Funnel plot of current meta-analysis. [file 12888_2019_2269_MOESM2_ESM.tif]
